# Factors influencing the re-emergence of plague in Madagascar

**DOI:** 10.1042/ETLS20200334

**Published:** 2020-12-01

**Authors:** Jennifer Alderson, Max Quastel, Emily Wilson, Duncan Bellamy

**Affiliations:** 1 Kennedy Institute of Rheumatology, University of Oxford, Oxford OX3 7FY, U.K.; 2 Nuffield Department of Medicine, University of Oxford, Oxford OX3 7FZ, U.K.; 3 School of Clinical Medicine, University of Cambridge, Cambridge CB2 0SP, U.K.; 4 The Jenner Institute, University of Oxford, Oxford OX3 7DQ, U.K.

**Keywords:** climate, drug resistance, infection, insecticide resistance, re-emergence, *Yersinia pestis*

## Abstract

Plague is an infectious disease found worldwide and has been responsible for pandemics throughout history. *Yersinia pestis*, the causative bacterium, survives in rodent hosts with flea vectors that also transmit it to humans. It has been endemic in Madagascar for a century but the 1990s saw major outbreaks and in 2006 the WHO described the plague as re-emerging in Madagascar and the world. This review highlights the variety of factors leading to plague re-emergence in Madagascar, including climate events, insecticide resistance, and host and human behaviour. It also addresses areas of concern for future epidemics and ways to mitigate these. Pinpointing and addressing current and future drivers of plague re-emergence in Madagascar will be essential to controlling future outbreaks both in Madagascar and worldwide.

## Introduction

Plague is a zoonotic disease caused by *Yersinia pestis*, a bacterium that usually survives in a rodent host and flea vector. Transmission to humans is initially from the flea, causing bubonic or — rarely — septicaemic plague, but the disease can become propagated from human to human via airborne droplets as pneumonic plague [1]. Bubonic plague manifests in buboes (enlarged lymph nodes) and has a case fatality rate of 20.8% with treatment or 40–70% without; pneumonic plague causes lung symptoms including shortness of breath and cough, with case fatality rates of 60.5% with treatment and 100% without [2,3]. The plague has affected humans for millennia, causing three major pandemics in the last 2000 years involving 200 million deaths [4], and has been endemic in the Madagascan highlands above 800 m altitude since the 1920s, showing a seasonal pattern of outbreaks [5–7].

**Figure 1.**
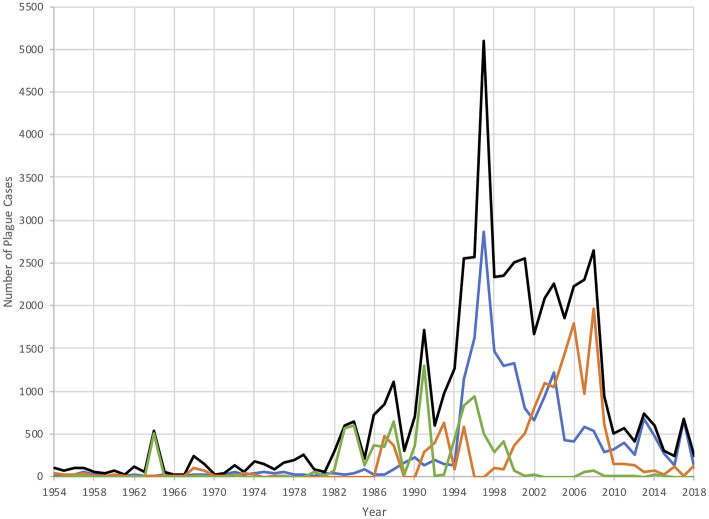
Plague cases from 1954 to 2018. Number of plague cases from 1954 to 2018; in the whole of Africa (black), in Madagascar (blue), in the Democratic Republic of Congo (DRC) (orange), and in the United Republic of Tanzania (URT) (green) [8–11]. Please note that reported numbers of plague cases in 2017 are inconsistent between sources — here the figures from the WHO 2019 report have been used [11].

Factors governing these outbreaks are complex, involving changes in rodent populations, flea populations and the interactions of these with humans as well as the bacterium itself. During the 1950s, antibiotics, insecticides, and improved standards of living contributed to controlling the disease in Madagascar at fewer than 50 cases per year for the next three decades [7]. However, cases began to increase in the 1990s and annual case numbers since 2000 vary between 100 and 700 (Figure 1). Other parts of Africa including the Democratic Republic of Congo and Tanzania have also experienced spikes in cases (Figure 1). In 2006, the WHO described the plague as re-emerging in Madagascar and the world [12] due to the increase in incidence and geographical spread of cases [13].

This review will explore factors implicated in plague re-emergence in Madagascar, including changes in the environment, weather and climate, changes in host and vector species, socioeconomic changes, resistance of vector species against insecticides, and resistance of hosts against disease. It will also address antibiotic resistance in *Y. pestis*. As there is no licensed vaccine currently recommended by the WHO [14], greater understanding of these factors will enable preparation for and prevention of future outbreaks, and may show parallels with similar re-emerging diseases.

## Rodents, fleas and *Y. pestis*

**Figure 2.**
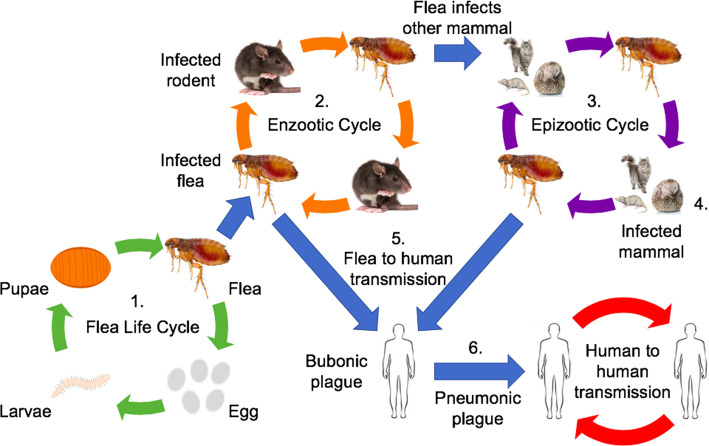
Diagram of plague transmission. 1. Flea life cycle: Fleas lay their eggs in the moist soil of rodent burrows; larvae feed on rodent faeces, and develop to form pupae and later fleas. Adult fleas parasitise rodents and can become infected with *Y. pestis*. 2. Enzootic cycle: Rodents act as hosts and reservoirs for *Y. pestis*, vectored by the fleas *X. cheopis*, *X. brasiliensis*, and *S. fonquerniei*. 3. Epizootic cycles occur when fleas infect mammals which are not the natural hosts of *Y. pestis*. 4. These newly infected mammals may spread plague to new areas and, lacking resistance, die quickly from *Y. pestis*. 5. Humans may become infected via the bite of a flea originating from an infected rodent or other mammal. 6. Bubonic plague may develop into pneumonic plague by affecting the lungs to induce a cough – this generates respiratory droplets containing *Y. pestis* which are inhaled by other individuals, resulting in direct transmission between people [1,15].

In Madagascar, *Y. pestis* is transmitted by the flea species *Xenopsylla cheopis*, *Xenopsylla brasiliensis*, and *Sinopsyllus fonquerniei* which parasitise the rodents *Rattus rattus* and *Rattus norvegicus* [1,15]. After ingestion during a blood meal from an infected mammal, the bacterium forms a biofilm blocking the flea proventriculus [4,16]. The blockage bursts when the flea next feeds, injecting bacteria into the dermis of a new host. This forms the enzootic cycle in Figure 2. Humans can enter the epizootic cycle if bitten by an infected flea, developing bubonic plague which can become pneumonic, spreading between humans via respiratory droplets.

## El Niño Southern Oscillation (ENSO), the Indian Ocean Dipole (IOD) and plague

Large-scale climate events affect not only localised weather but also the incidence of many infectious diseases. Here, we address climate events specifically implicated in plague.

### Climate influences

ENSO events are characterised by warm, dry El Niño periods and cooler, wetter La Niña periods due to changes to sea surface temperatures and air pressure in the Pacific Ocean. ENSO is a global climate driver and is recognised to affect numerous infectious diseases. ENSO events may maintain strong positive or negative correlations with plague incidence [17].

Changes to Indian Ocean surface temperatures create the IOD. Positive events are characterised by warmer water in the western Indian Ocean and cooler water in the east, whereas negative events have cooler western water and warmer eastern. Positive events create warm wet weather in Madagascar; negative ones create cooler drier conditions [17,18]. IODs can mitigate or contribute to the effects of ENSO on plague.

**Figure 3.**
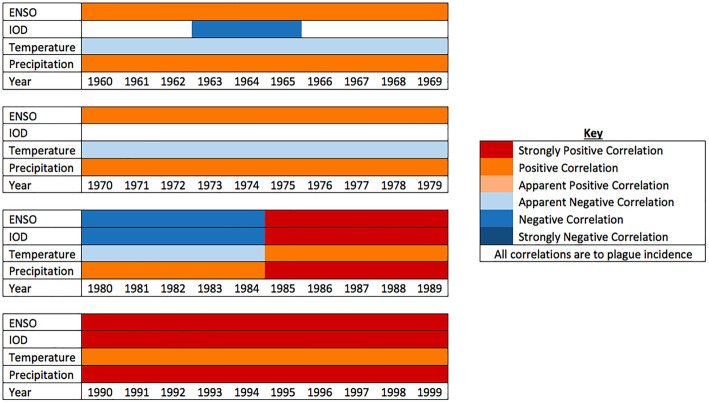
Correlations between climatic factors and plague. The relationship between plague incidence and climate are complex and further explored in the following section. Some years a positive or negative correlation appears between plague and climatic factors; other years have none, and not all years have ENSO or IOD events. Note the strong positive correlations of plague with all climatic factors in the 1990s [17].

Kreppel et al. [17] demonstrated a complex relationship between ENSO, IOD and Madagascan plague, using wavelet analysis of data from 1960 to 2010. This is summarised in Figure 3.

This statistical analysis of Madagascan plague incidence and climate describes a strong positive correlation between ENSO and plague incidence in the mid-1990s [17]. IOD, temperature and precipitation were also each positively correlated with plague incidence over the same period (Figure 3). A similar comparison of ENSO, Pacific Decadal Oscillation (PDO) and plague incidence in the US [19] also found that positive ENSO events combined with positive PDO events and precipitation correlated with higher plague incidence.

ENSO-related temperature changes occurring during cold dry months between May and September do not favour plague transmission. ENSO events in this season account for the negative correlation in the 1980s [17]. In contrast, the 1990s saw an unusually strong ENSO event that persisted over 5 years [20] and were at a time of strong positive correlation between ENSO and the IOD, creating a warm wet local climate. They also marked a notable increase in annual cases for Madagascar: plague cases, previously always below 200, were 1147 in 1995 and 2863 in 1997 (Figure 1), the year of the strongest El Niño event and IOD in the analysis [17]. The statistical analysis of ENSO, IOD and plague correlation between 1960 and 2000 shows the climate events taking place were very likely a driver of the 1995 and 1997 spikes in plague [17]. Since 2000, Madagascar has maintained ∼74% of annual cases worldwide [21] with an average of 640 cases per year (Figure 1).

A model developed for plague cases in Madagascar between 1980 and 2007 included climatic factors in addition to others such as population density and elevation [22]. While the association of plague with precipitation was found to be weak, a cumulative effect of temperature and elevation on plague was identified and this model showed good correlation between predicted and actual cases. To understand how climate phenomena may affect plague incidence we examine the effects of weather, temperature and precipitation on host, vector and bacterium.

### Downstream effects on rodents, fleas and *Y. pestis*

There are complex relationships between the environment, *Y. pestis* and its vectors and hosts. Ecological events affect these by various mechanisms, summarised in Table 1.

**Table 1.** Effects of climate conditions on host, vector and *Y. pestis*

	Rats	Fleas	*Y. pestis*
Low temperature (<10°C)	Lack of food = ↑ rodent mortality	↑↑ vector mortality from *Y. pestis* infection	Faster bacterial growth ↑ expression of proteins for transmission (e.g. Hms for biofilm formation) ↑ expression of proteins for survival within flea (e.g. Pms for iron absorption) ↓ expression of proteins for mammalian immune evasion (e.g. Type 3 secretion system, V antigen)
High temperature (>27°C)	Droughts (causing food shortages) = ↑ rodent mortality and ↑ proximity to humans while searching for food	↓ biofilm blockage of flea proventriculus = bacteria are lost from flea gut, causing ↓ transmission	No biofilm formation >26°C (Hms protein does not form) ↓ bacterial growth >35°C.
Precipitation and humidity	↑ spring rainfall = more food, causing ↑ rodent population 1–2 years later Flooding = ↑ rodent death and ↑ proximity to humans (including movement into homes)	Humidity of 50% essential for larval development >80% humidity = ↑ bite rate. >95% humidity = ↑ chance of fungi destroying larvae and eggs Flooding = ↑ egg and larval destruction	

The immature stages of flea development (Figure 2) occur in the soil of the host burrow and are directly affected by humidity, precipitation and temperature; warm moist conditions are optimal for flea development. Note that climatic factors have effects on multiple aspects of the plague cycle, which can also be affected by events in previous years. These complexities mean that prediction of human plague cases cannot be easily inferred from individual factors [17, 23, 24].

During the mid-1990s a positive IOD preceded a plague outbreak by approximately 9 months [17]. Increased rainfall was the first effect of the IOD, followed by warmer temperatures. This likely led to an abundance of food for rodents before the mating season, and may have created ideal conditions for flea development (Table 1). The combination with an unusually intense ENSO period led to the significant increase in cases in 1995.

Two years of warm wet weather followed, enabling rodent population expansion, with increasingly strong ENSO events 1997, in particular, is an excellent demonstration of the effects climate events can have on plague both immediately and in subsequent years, due to the varying time scales of the impacts on vectors and hosts. It stresses the importance of the role serial and tandem climate events play, and indicates the need for consideration of climate factors over multiple years when predicting and preparing for plague epidemics.

## Resistance

There are three main forms of resistance affecting plague epidemiology. The resistance of rodents to plague manifestation forms a disease reservoir, while insecticide resistance of fleas enables increased propagation of disease through the rodent population and more frequent transmission to humans. These increase the likelihood of small outbreaks of disease in humans. In comparison, antibiotic resistance in *Y. pestis* does not increase the likelihood of transmission to humans but gives the potential for these outbreaks to spread uncontrolled by increasing human to human transmission.

### Rodent resistance to plague

Resistance or susceptibility of rodents to plague varies based on their species and location. Rodents with low resistance to plague are more likely to die quickly after infection, causing their fleas to jump to another host which may be a human [25,26]. However, rodents with higher resistance act as a disease reservoir for longer [26]. A comparison of rodents from different locations in Madagascar is summarised in Table 2.

**Table 2.** A comparison of rodent species and their resistance to *Y. pestis* depending on the location they were captured from [26]

Rodent species	Origins	LD_50_/cfu
*R. norvegicus*	Albino laboratory rat	<10
*R. norvegicus*	Madagascan ‘plague-free’ areas	<100
*R. rattus*	Madagascan ‘plague-free’ areas	<100
*R. norvegicus*	Antananarivo	10^3^
*R. rattus*	Antananarivo	10^5^

Resistance or susceptibility of different rodent species to disease can be quantified by using a subcutaneous injection of bacteria to simulate infection via flea bite, followed by measuring the number of bacterial colony forming units (cfu) required to kill half the rodents injected (LD_50_). A higher LD_50_/cfu demonstrates greater resistance to plague.

### Flea resistance to insecticides

Insecticides are commonly used to control plague in Madagascar, reducing the number of vectors and therefore transmission between hosts and to humans. These are applied via indoor residual spraying, which seems to have a negligible environmental impact [27,28]. However, use in urban areas has led *X. cheopis* resistance being reported, first for DDT [29] then for dieldrin, malathion, fenitrothion, and propoxur [30,31], deltamethrin [32], and recently to alphacypermethrin, etofenprox, and lambda-cyhalothrin, which are pyrethroids like deltamethrin, are pyrethroids but have never been used to control vector populations in Madagascar [33].

The few insecticide resistance studies carried out for *S. fonquerniei* and *X. brasiliensis* have reported DDT, malathion and pyrethroid resistance in Madagascar for the former [31], and DDT resistance for the latter in Tanzania [31,34]. This suggests there may be widespread insecticide resistance in Madagascar, but requires verification by further studies.

### *Y. pestis* resistance to antibiotics

The site of a flea bite offers little opportunity for bacteria to exchange genetic material and/or resistance factors. Although *Y. pestis* acquiring drug resistance plasmids is mainly limited to the flea's gut bacteria from feeding on mammals with bacteraemia [35], these conditions can be favourable for their exchange [36,37]. Unsterile sites including the gastrointestinal tract of predatory animals after infected meat consumption [38] and less common pharyngeal, oesophageal and enteric forms of plague [39] provide further opportunities for unrelated bacteria to congregate with *Y. pestis* and exchange drug resistance plasmids.

While the first-line antibiotic for the treatment of plague is streptomycin as recommended by the WHO, various other antibiotics including tetracyclines, chloramphenicol and fluoroquinolones are also used for patient treatment and contact prophylaxis [40,41]. Regular use of such a range of broad-spectrum antibiotics theoretically increases the risk of antibiotic resistance in *Y. pestis* and other bacterial flora [42]. However, the only known occurrences of drug-resistant plague in humans were in Madagascar in 1995: strain 16/95 from a patient in Ambalavao showed streptomycin resistance, whereas strain 17/95 from a patient in Ampitana had multidrug resistance [35,43]. Neither of the resistance factors found on plasmids unique to each strain (pIP1202 and pIP1203, respectively) [35] were the same, and the two villages were geographically distant.

Since 1995, resistant *Y. pestis* has not been identified in human isolates [10,44], but a resistant *Y. pestis* strain has been found in *R. norvegicus* in Antananarivo [45] and elsewhere in the world [46]. As a result, while antibiotic resistance does not appear to be driving the re-emergence of the Madagascan plague, it may become more important in the future and would benefit from continued monitoring.

## Other drivers of re-emergence

A requirement for plague re-emergence is human contact with infected rodent and flea populations. Different rodents have different preferred habitats: while both *R. norvegicus* and *R. rattus* are nocturnal, *R. norvegicus* usually lives in burrows, making sewers a suitable habitat, whereas *R. rattus* typically lives in trees and is frequently found in the thatch of housing [1]. *R. norvegicus*, therefore, has lower rates of contact with humans than *R. rattus* and causes less frequent plague human outbreaks [37].

Proportions of *R. rattus* and *R. norvegicus* vary across Madagascar. Since the late 1950s urbanisation and improved housing quality with concrete roofs favoured *R. norvegicus* over *R. rattus*, causing increased proportions of *R. norvegicus* in urban communities. In Antananarivo in 1979, 80% of rodents captured were *R. norvegicus* compared with 20% *R. rattus* [37]; by 2001, 100% were *R. norvegicus* [26]. Lower contact rates between humans and *R. norvegicus* coupled with higher resistance of urban rats to *Y. pestis* has led to a comparatively low incidence of plague in urban areas since [37].

In contrast, *R. rattus* is often found in rural settings, living in houses which retain traditional thatched roofs and the surrounding fields and forests [1,47]. Deforestation around these areas enables *R. rattus* to travel deeper into surrounding areas and contact sylvatic plague reservoirs, while also allowing sylvatic species that have lower resistance to plague to move closer to human communities [37]. *X. cheopis* lives on both *R. rattus* and *R. norvegicus* but only on rats living in close proximity to humans [1], so *R. rattus* is likely to acquire sylvatic vectors carrying *Y. pestis* from wild animals it encounters [37]. *Y. pestis* is also particularly likely to be transmitted to humans in these areas as the people cultivating newly deforested land are more likely to be migrant workers with poorer living conditions and increased contact with rodent hosts [37]. It is possible the human flea *Pulex irritans* may play a role in *Y. pestis* transmission between humans once an outbreak begins, as noted by a study examining the 2012 and 2013 Madagascan outbreaks [48].

Social inequality and political unrest are also implicated in increasing contact between rodents and humans. *R. rattus* is more often found in deprived neighbourhoods of urban communities, and can be driven into temporarily closer proximity to humans by events such as flooding [37]. In 1978–1979 a significant reduction in the socioeconomic condition of the population occurred simultaneously to the reappearance of plague in Antananarivo [26]; political unrest in the 1980s and 1990s affected public health services including surveillance measures and insecticide programmes, coinciding with another rise in cases [37]; discontinuation of plague surveillance in Antananarivo from 2006 due to financial constraints likely contributed to the subsequent reappearance of plague there [3].

Cultural behaviours also affect plague epidemiology. Malagasy traditional burials involve ritual exhumation and rewrapping of corpses, and chains of pneumonic plague transmission from funerals have been established [37,49]. There is governmental guidance advising against the exhumation of plague victims for 7 years after death, but this is not always followed by the Malagasy people. After the 2017 outbreak, the WHO and UNICEF developed a new burial protocol in conjunction with the local population to increase safety [50]. It should also be noted that a plague diagnosis can carry significant stigma for individuals and whole village communities in Madagascar, which may impede progress in controlling outbreaks. During the 2017, outbreak the Red Cross warned this stigmatisation risked contact tracing due to individuals being unwilling to come forward for testing [51] and emphasised the importance of anti-stigma activity at both community and national levels in their Final Action Report [52].

While plague cases have previously been linked to handling wild animals [53] and consumption of bush meat [38], this is rare and has not yet been reported in Madagascar.

Madagascar's unique geography continues to pose challenges to controlling outbreaks. Since plague most often arises in rural highland areas where it is transmitted by fleas on *R. rattus* [54], a combination of remoteness and local banditry can slow access to these regions [55] and field samples can experience long delays in transport to laboratories [3]. Misdiagnosis and lack of understanding of plague can also be a challenge in controlling outbreaks in regions that typically experience very few cases [3].

There are several types of test to diagnose plague, including bacterial culture, molecular tests and rapid diagnostic tests. It is important to note these tests are of differing utility in bubonic and pneumonic plague: the use of a molecular test less specific for pneumonic plague in the 2017 outbreak, which had an unusually high proportion of pneumonic cases, led to initial overestimates of confirmed plague cases before samples were retrospectively tested with a more specific but potentially less sensitive molecular test for pneumonic plague [21]. Notably, the F1 rapid diagnostic test which can be carried out at the bedside, negating some of the geographical issues above, has high sensitivity but does not give the same information on *Y. pestis* strain or antibiotic resistance as bacterial culture [56].

## Vaccines

There are many potential strategies to mitigate future plague outbreaks, including improving living conditions, vector eradication, and timely antibiotic treatment. However, an invaluable long-term strategy would be the appropriate use of an effective vaccine, as previously demonstrated for many other infectious diseases including measles, pneumococcal infection, yellow fever and cholera [57–59].

Two vaccines are currently available for plague: an inactivated vaccine used in the Western world and a live attenuated vaccine used in former USSR countries and former French colonies [60]. However, both have significant issues. The inactivated vaccine requires multiple doses, efficacy evidence is circumstantial, and it appears to protect against bubonic but not pneumonic plague [60]; the live attenuated vaccine does not have reliable safety and efficacy profiles [60] and the bacterial strain used is not uniform [61]. The WHO does not currently recommend vaccination except for high-risk workers, such as laboratory personnel and healthcare workers [62].

The 2018 WHO plague workshop summarises the 17 vaccines currently in development, including subunit, viral vector-based, bacterial vector-based and live attenuated vaccines [14], and prospective vaccines are examined in more detail by Sun and Singh [61]. The F1 capsule protein and V antigen (which is encoded by LcrV and binds human Toll-like receptor 2) appear to protect against infection in mouse models [60] and are targets of the vaccine closest to market: the US FDA granted the rF1 V vaccine, currently in phase II clinical trials, Orphan Drug status in 2017 [63]. However, this vaccine has outstanding issues including the existence of LcrV polymorphisms and virulent non-encapsulated *Y. pestis* strains not expressing the F1 protein [64], as well as differing indications of the effectivity of antibodies against V antigen at preventing pneumonic plague [60,61]. Sun and Singh [61] propose the most effective form of the vaccine may be heterologous primer-boost, combining a subunit vaccine with a live attenuated or vectored vaccine.

The 2018 WHO workshop concluded that the WHO currently continues not to recommend any plague vaccine. Concerningly, it stresses the potential need to trial a vaccine during a future plague outbreak, which may be the only opportunity to gather the necessary evidence for wider use of the vaccine but cannot take priority over control of the outbreak [14].

## Discussion

Plague undergoes regular cycles of epidemics in Madagascar due to vector transmission following contact between rat hosts and human communities. Since 2006 the WHO has categorised it as re-emerging due to the increase in incidence and geographical spread of cases [12,13]. Many factors affecting this are longstanding, from poor housing conditions to deforestation enabling contact with sylvatic reservoirs and vectors. Flea resistance to insecticides has been increasingly reported in Madagascar since the 1960s [29–33]. However, the unique ENSO and IOD events and political and socioeconomic deterioration in the 1990s coincided with a rise in plague cases that has persisted.

Climate has relatively recently become of interest as a plague driver, but the statistical analysis of ENSO and IOD events of the 1990s in relation to plague suggests its effect can be significant. In the U.S.A., a similar relationship of ENSO and the PDO also exerts effects on plague incidence [19,23,65]. Events of the 1990s demonstrate both the immediate and longer-term effects climate can have on plague, with repeated climatic events eliciting a crescendo effect on plague cases over time. Considering these climatic events together and modelling their effects may become a means of predicting and preparing for plague outbreaks in the future as the global climate continues to change.

Current strategies used to control plague in Madagascar include prevention of human infection by using insecticides to destroy flea vectors, antibiotic treatment of cases, encouragement of safe burial practices to prevent pneumonic transmission, and case surveillance to monitor outbreaks. Achieving these requires overcoming various challenges, not least the logistical difficulties of accessing geographically isolated populations in potentially unstable regions [55] and the cultural stigma associated with the plague. While these strategies have historically been successful in controlling outbreaks, rising insecticide resistance [3] poses an increasing threat and the identification of antibiotic-resistant *Y. pestis* strains remains a concern. The rise in cases and repeated epidemics since 1990 may require new strategies.

The proportion of pneumonic cases has increased over the last two decades due to the deteriorating Malagasy healthcare system failing to treat bubonic cases in a timely manner [3]. Another significant pneumonic plague outbreak in Madagascar in the future is not unlikely. However, there are various measures that could reduce the likelihood of this, often reflecting strategies used to control other infectious diseases.

Transmission of plague to humans has previously been reduced in Madagascar by improving living conditions and minimising contact between rodents and humans [37]. This has also been effective elsewhere in reducing diseases including leptospirosis, a bacterial disease spread by contact with *R. norvegicus* [66], and malaria, spread by mosquito vectors [67]. Transmission could reasonably be minimised by reducing deforestation rates to reduce contact between humans and rodents, reducing socioeconomic inequality and maintaining good quality rat-proof housing, and finding new means of eradicating flea vectors. Unfortunately, these are all logistically difficult, as they operate on a wide scope and would require significant Malagasy political, social, and financial commitment.

Living conditions remain important during the pneumonic propagation of outbreaks. Tuberculosis also spreads via droplet transmission and is well-recognised to have the higher transmission in poorer, overcrowded living conditions [68]; it is reasonable to suspect this is similar for pneumonic plague. There are also similarities with other airborne diseases: as for COVID-19 [69], the transmission of pneumonic plague is thought to be reduced by the wearing of masks and use of social distancing [70].

However, long-term control of infectious disease is often dependent on the development of a safe and effective vaccine, as seen for the airborne viruses measles, mumps and rubella globally [57] and the bacterial disease cholera in Asia [59]. While various vaccines against *Y. pestis* are currently in development, challenges include the existence of virulent *Y. pestis* strains that do not express the same target proteins, poor immunogenicity of subunit vaccines, and the difficulty of structuring clinical trials to prove safety and efficacy. Even after vaccine approval, immunisation of all at-risk individuals would require a significant Malagasy public health movement.

Finally, potential measures that could be implemented include surveillance of Madagascan *Y. pestis* strains for antibiotic resistance although, as mentioned previously, antibiotic resistance is rare in *Y. pestis* and has not caused a significant issue in previous outbreaks. Again, predictive modelling of ENSO and IOD events combined with other factors affecting plague could also enable Malagasy authorities and external organisations to anticipate outbreaks, plan resource allocation, and put in place targeted prevention measures.

## Summary

- Plague has been endemic in Madagascar since the early 1920s with significant resurgence in the 1990s. In 2006, plague was categorised by the WHO as a re-emerging disease in Madagascar and the world.
- Historically plague has been reduced by improved living conditions and indoor residual spraying of insecticides to kill flea vectors. Outbreaks have been controlled by rapid identification and treatment of cases with antibiotics, anti-stigma efforts, and encouragement of safe burial practices of plague victims.
- Likely factors contributing to the re-emergence of plague in Madagascar include climatic events and socioeconomic deprivation, as seen in the 1990s, and increasing vector resistance to insecticides.
- Areas of concern for future outbreaks include regular recent outbreaks, the deterioration of Malagasy public health measures accompanied by the increasing proportion of pneumonic cases in recent outbreaks, and the potential effects of climate change on the incidence and geographical spread.
- This review suggests many steps to minimise the risk of future large-scale plague epidemics: minimisation of contact between humans and rodent hosts of *Y. pestis*, use of social distancing and masks to control pneumonic outbreaks, and development and distribution of a safe and effective vaccine. Other strategies could include monitoring of Madagascan *Y. pestis* strains for antibiotic resistance and predictive modelling of plague epidemiology based on climatic and other factors.

## Open access

Open access for this article was enabled by the participation of University of Oxford in an all-inclusive *Read & Publish* pilot with Portland Press and the Biochemical Society under a transformative agreement with JISC.

## Abbreviations

ENSO: El Niño Southern Oscillation
PDO: Pacific Decadal Oscillation
IOD: Indian Ocean Dipole
